# Virtue and care ethics & humanism in medical education: a scoping review

**DOI:** 10.1186/s12909-021-03051-6

**Published:** 2022-02-26

**Authors:** David J. Doukas, David T. Ozar, Martina Darragh, Janet M. de Groot, Brian S. Carter, Nathan Stout

**Affiliations:** 1 Department of Family and Community Medicine, James A. Knight Chair of Humanities and Ethics in Medicine, Program in Medical Ethics and Human Values, Tulane University School of Medicine, 1430 Tulane Ave, #8033, New Orleans, LA USA; 2 Loyola University, Chicago, USA; 3 Georgetown University, Washington, USA; 4 University of Calgary, Calgary, CA Canada; 5 University of Missouri-Kansas City, Kansas City, USA

**Keywords:** Virtue ethics, Care ethics, Humanism, Medical education, Medical ethics

## Abstract

**Purpose:**

This scoping review explores how virtue and care ethics are incorporated into health professions education and how these factors may relate to the development of humanistic patient care.

**Method:**

Our team identified citations in the literature emphasizing virtue ethics and care ethics (in PubMed, NLM Catalog, WorldCat, EthicsShare, EthxWeb, Globethics.net, Philosopher’s Index, and ProQuest Central) lending themselves to constructs of humanism curricula. Our exclusion criteria consisted of non-English articles, those not addressing virtue and care ethics and humanism in medical pedagogy, and those not addressing aspects of character in health ethics. We examined in a stepwise fashion whether citations: 1) Contained definitions of virtue and care ethics; 2) Implemented virtue and care ethics in health care curricula; and 3) Evidenced patient-directed caregiver humanism.

**Results:**

Eight hundred eleven citations were identified, 88 intensively reviewed, and the final 25 analyzed in-depth. We identified multiple key themes with relevant metaphors associated with virtue/care ethics, curricula, and humanism education.

**Conclusions:**

This research sought to better understand how virtue and care ethics can potentially promote humanism and identified themes that facilitate and impede this mission.

## Background

Health care education in the last two decades has led many to grapple with the means of better understanding professionalism in medical education [16]. The Project to Rebalance and Integrate Medical Education (PRIME) [29, 33–35] framed those aspects of knowledge and skills that lead to professionalism formation. PRIME characterized a construct for professionalism in which medical ethics (including both virtue and care ethics), fine arts, medical history, and narrative were asserted as requisite in the knowledge domain needed for resultant humanistic behavior. Understanding how these approaches to ethics inform health care curricula and humanism, we can examine the connections between them that others have postulated [30, 46, 52, 54]. This project aims to identify published studies that define virtue and care ethics, then use these concepts in medical curricula, and identify their association with humanistic behavior.

We seek to identify health care curricula using virtue and care ethics and how humanistic behavior is thereby promoted. Virtue ethics is one of three major ethical theories on how one should respond to moral quandaries. Virtue ethics emphasizes how optimizing those aspects of moral character will predispose one to behave in an ethical manner and has been frequently described as core to professionalism [47]. The other ethical theories commonly used in medical education are Deontology, from which classical duties of Respect for Persons and Justice in contemporary bioethics are grounded, and Consequentialism, which evaluates the outcomes of one’s actions and includes the principles of Beneficence and Non-maleficence [27]. Virtue ethics includes moral reasoning, altruism, beneficence, honesty, and integrity and is thought to motivate professional behavior when enacted through practice [41].

Care ethics at its core is relational [40, 51] and thus differs from virtue ethics where care may be a virtue that individuals seek to enhance [42]. The concept of care is both core to and distinct from care ethics. In the context of healthcare, care ethics acknowledges that caring occurs in unequal relationships, in which one is more vulnerable and receives care [31]. Overall, care is described as co-constituted [42], with moral reasoning occurring in the context of relationships, incorporating emotion. Care ethics was developed, in part, in opposition to [39], or in some authors, as a crucial complement to, deontological and consequentialist theories [36, 45, 50].

The Gold Foundation has had a pervasive educational presence in medical schools, with chapters influencing education in 172 North American and Caribbean allopathic and osteopathic medical schools. For an operational definition of humanism, we cite that offered by the Arnold P. Gold Foundation: *“Humanism in healthcare is characterized by a respectful and compassionate relationship between physicians, as well as all other members of the healthcare team, and their patients. It reflects attitudes and behaviors that are sensitive to the values and the cultural and ethnic backgrounds of others”* [38]. The goal of this study was to better understand how virtue and care ethics were used in medical curricula to advance the valuable role of humanism in health care. Our search was based on authors using the term “humanism.”

As the Liaison Committee on Medical Education (LCME) requires that all medical schools have medical ethics and human values as part of their undergraduate curricula, this scoping review is poised to identify how such current virtue and care ethics teaching is put into effect with varying curricula, and then connected with learner humanistic behavior [44]. Our effort aims to identify curricular implementation of virtue and care ethics and their contribution to learner humanism, as there has not been a prior scoping review on this topic. Hence, our Research Question was formulated as: *How has virtue and care ethics been implemented in medical curricula, and how is it related to learner humanism in the medical literature?*

## Method

We emulated the Scoping Review method articulated by Levac [43] to evaluate and collate information found on virtue and care ethics in medical education, then analyze relevant themes, and make note of significant metaphors that then lend themselves to our research question [26, 28, 43]. We first identified our research question, identified relevant citations, extracted data from the literature, and collated and summarized our results. We examined publications with three areas of interest: virtue and care ethics, their incorporation into medical curricula, and any description of humanistic behavior as an outcome. The search strategy was developed with the assistance of a university librarian (MD) and adapted to each database. We conducted a search in PubMed with the following:

- ((“Virtues” [Mesh] OR virtues OR “virtue ethics” OR Caring [Mesh] OR “care ethics”) AND (“Humanism”[Mesh] OR humanism) AND (Education, medical [Mesh] OR “medical education” OR professionalism)).

**Fig. 1:**
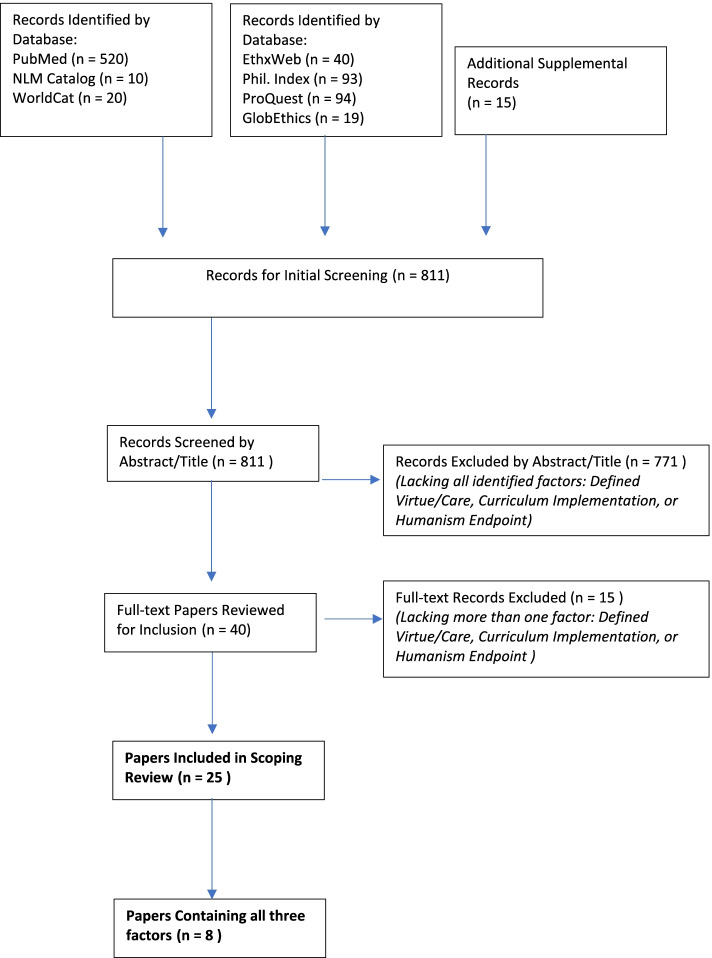
Search Algorithm

Our team identified the literature in ethics highlighting those aspects with an emphasis on Virtue Ethics and Care Ethics, first in PubMed (1972 -). Despite using the Boolean “OR” for keywords and PubMed Mesh terms for maximum inclusivity, we noted a grave paucity of articles with our first PubMed search query. Thereafter, we employed PubMed’s unique database feature of *full text searching of our search terms within the abstracts* of each identified citation. As shown in Fig. 1, we broadened our search in the following databases NLM Catalog (1993 -), World Cat (1997 -), EthicsShare (1984 -), EthxWeb (1974–2011), Globethics.net (2004 -), Philosopher’s Index (1986 -), and ProQuest Central (1986 -), which incrementally added to the critical mass of resultant articles (by MD). Searches were initiated with PubMed on December 19, 2017, with subsequent stepwise additions utilizing the other identified databases through October 3, 2018.

Our exclusion criteria consisted of: *non-English articles, those not addressing the use of virtue and care ethics toward humanism in medical curricula, and those not addressing aspects of character in health ethics*.

We read and analyzed eligible articles in a stepwise fashion, considering iteratively the following three requisite considerations “Does this paper…:”

1. Have a well-accepted definition of virtue or care ethics? (We characterized each of these broadly and included any paper that prioritized the development of character or of caring relationships in their conception of medical ethics/values.)
2. Describe how virtue or care ethics are implemented in a healthcare curriculum?
3. Describe curricular efforts that contribute to patient-centered caregiver humanism?

Five hundred twenty articles were identified and evaluated using PubMed, with other ancillary databases yielding 246 additional articles, and 15 supplemental papers considered through citation referral yielding a total of 811 papers being screened. Team members DD, DO, MD, JdG, and BC evaluated the titles and abstracts of all papers. An initial review found 88 papers having at least one of the three sought factors. Next, papers were included only if *at least* ***two*** *of the three factors were present: definition, curricular inclusion, and evidence of humanistic behavior as an outcome*. The resultant 40 relevant papers were subjected to intense analysis by each team member. Each paper was scored on a four-level relevance scale (none, minimal, moderate, and high). Inclusion was based on the majority of team members scoring the article as moderate or high. Each team member identified relevant themes and metaphors that were then discussed by conference call pertaining to virtue/care ethics, curricula, and humanism education. Differences in scoring of these 40 papers were minimal and negotiated by all team members via conference call, until we achieved consensus on 25 papers.

## Results

As depicted in Fig. 1, a total of 811 papers were evaluated, with subsequent Abstract full text search analysis identifying 25 papers deemed relevant in at least two of the three domains, and eight papers had all three factors under consideration. The factor that was most often missing was evidence of an implemented curriculum — we have indicated in bold on our Resultant Publication list those eight papers containing all three factors. Our practical decision to conduct detailed analysis of all 25 papers was based on the relative lack of articles containing all three factors, and it was noted that 17 articles lacking an *implemented* curriculum either *proposed* a curriculum or *argued for* curricular change.

The distinctive quality of these eight papers is that each lays a foundation of the conceptual framework of either virtue or care ethics and builds upon that basis with a curriculum that identifies humanistic outcomes. These papers emphasize multiple venues for virtue or care ethics — in undergraduate and graduate medical education, as well as in faculty development promoting mentorship — and as such, provide instructive templates towards future curricular development in this area.

The 25 articles range in date from 1994 to 2017, starting in proximity to when Gold Foundation Humanism efforts to foster humanism began and flourished, and when professionalism as a competency was introduced in US medical education assessment with one publication cluster noted in 2005–2009, and another cluster in 2015–2016. The journal *Academic Medicine* had the largest number of publications, with the Journal of Medical Ethics following, and then a distribution between other medical ethics and health service journals and publishing houses. We next proceeded to identify relevant themes.

### Themes

DD and NS developed a draft set of themes that emerged and were negotiated on multiple readings of the 25 papers cited, which were subsequently refined with JdG and BC, resulting in nine distinctive themes and four side issues in these papers (Table 1). These themes were: Altruism, Development of Virtuous Traits, Relationships, Dissonance/Virtue versus Principlism, Care as a Virtue, Praxis in Humanistic Behavior, Role Modeling, Pedagogy, and The Culture of Medicine/The Hidden Curriculum. Additionally, there were four significant *Noteworthy Considerations* that were identified and were coherent but not centrally on point as a theme of cultivating virtue/care ethics and humanistic behavior (Table 2). These considerations consisted of Professionalism/Professional Formation, Narrative, the Use of Learning Communities, and the Need to Prevent Burnout.

**Table 1:** Themes

Altruism	Development of Virtuous Traits	Care as a Virtue	Pedagogy	Role Modeling	Relationships	Praxis/Humanistic Behavior	Dissonance/Virtue vs. Principle	Culture/Hidden Curriculum
Coulehan (2005) [14]	Arnold et al. (2016) [1]	Benner (1997) [11]	Austin (2007) [9]	Austin (2007) [9]	Arnold et al. (2016) [1]	Arnold et al. (2016) [1]	Arnold et al. (2016) [1]	Coulehan (2005) [14]
Doukas (2003) [16]	Barilan (2009) [10]	Branch et al. (2009) [3]	Benner (1997) [11]	Branch et al. (2009) [3]	Benner (1997) [11]	Austin (2007) [9]	Benner (1997) [11]	Coulehan (2007) [13]
Brody & Doukas (2014) [12]	Bolsin et al. (2005) [2]	Coulehan (2007) [13]	Bolsin et al. (2005) [2]	Coulehan (2005) [14]	Branch et al. (2009) [3]	Barilan (2009) [10]	Cook et al. (2015) [4]	Brody & Doukas (2014) [12]
Irby & Hamstra (2016) [18]	Cook et al. (2015) [4]	Doukas (2003) [16]	Branch et al. (2009) [3]	Coulehan (2007) [13]	Kesselheim et al. (2015) [5]	Benner (1997) [11]	Coulehan (2005) [14]	Kotzee & Ignatowicz (2016) [20]
McCammon & Brody (2012) [23]	Coulehan (2005) [14]	Kotzee & Ignatowicz (2016) [20]	Cook et al. (2015) [4]	Osterberg et al. (2015) [6]	Madani et al. (2017) [22]	Bolsin et al. (2005) [2]	Coulehan & Williams (2001) [15]	McCammon & Brody (2012) [23]
Schaechter & Canning (1994) [7]	Coulehan (2007) [13]	Madani et al. (2017) [22]	Coulehan (2007) [13]		Osterberg et al (2015) [6]	Branch et al. (2009) [3]	Doukas (2003) [16]	
	Coulehan & Williams (2001) [15]	Osterberg et al. (2015) [6]	Gould (2002) [17]		Schaechter & Canning (1994) [7]	Cook et al. (2015) [4]	Brody & Doukas (2014) [12]	
	Doukas (2003) [16]	Schaechter & Canning (1994) [7]	McDougall (2013) [24]		Wald et al. (2015) [8]	Coulehan & Williams (2001) [15]	Irby & Hamstra (2016) [18]	
	Brody & Doukas (2014) [12]		Kopelman (1999) [19]			Gould (2002) [17]	Kotzee & Ignatowicz (2016) [20]	
	Irby & Hamstra (2016) [18]					Kesselheim et al. (2015) [5]	Leffel et al (2015) [21]	
	Kesselheim et al. (2015) [5]					Kopelman (1999) [19]	Madani et al (2017) [22]	
	Kopelman (1999) [19]					Kotzee & Ignatowicz (2016) [20]	McDougall (2013) [24]	
	Kotzee & Ignatowicz (2016) [20]					Madani et al. (2017) [22]	Toon (2007) [25]	
	Leffel et al (2015) [21]					McDougall (2013) [24]		
	Madani et al (2017) [22]					Osterberg et al (2015) [6]		
	McCammon & Brody (2012) [23]					Schaechter & Canning (1994) [7]		
	McDougall (2013) [24]					Toon (2007) [25]		
	Osterberg et al. (2015) [6]							
	Schaechter & Canning (1994) [7]							
	Toon (2007) [25]

**Table 2:** Noteworthy Considerations

Narrative	Prevention of Burnout	Learning Communities	Professionalism/Professional Formation
Arnold et al. (2016) [1]	Kesselheim et al. (2015) [5]	Branch et al. (2009) [3]	Barilan (2009) [10]
Austin (2007) [9]		Osterberg et al. (2015) [6]	Benner (1997) [11]
Coulehan (2005) [14]		Schaechter & Canning (1994) [7]	Bolsin et al. (2005) [2]
Coulehan (2007) [13]			Coulehan (2005) [14]
Osterberg et al. (2015) [6]			Coulehan (2007) [13]
Schaechter & Canning (1994) [7]			Coulehan & Williams (2001) [15]
Toon (2007) [25]			Doukas (2003) [16]
Wald et al. (2015) [8]			Brody & Doukas (2014) [12]
			Irby & Hamstra (2016) [18]
			Kopelman (1999) [19]
			Leffel et al (2015) [21]
			Madani et al. (2017) [22]
			McCammon & Brody (2012) [23]
			Osterberg et al. (2015) [6]
			Schaechter & Canning (1994) [7]
			Wald et al. (2015) [8]

The themes of Altruism, Development of Virtuous Traits, and Care as a Virtue are all related to the development of character in ethics within the educational process. The Altruism papers concentrated on perspectives that altruism is a prevailing virtue in medicine, often linked to the caring enterprise of healing [12]. Altruism was posited as running deeply such that this personal virtue can also be institutional, uniting the ideals of professional behavior [7]. Some propose that communities of learning should be cultivated, focusing on altruism to encourage humanism [23]. Altruism was considered a key virtue to counter negative influences in medical culture (discussed below) [14] Altruism also conceptually questioned: Can and should giving of oneself be so great as to allow self-sacrifice? [23].

The Development of Virtuous Traits theme appeared most commonly in our analysis, focusing on how virtue integrates health care values into the practice of medicine through a social contract [12]. The profession of medicine promotes virtues advancing professional conduct and patient healing [16]. Medical pedagogy in virtue and care ethics both identify aspects of character central to patient care [23]. Understanding virtue ethics may assist learners in better understanding ethical issues in healthcare, and thereby support development of humanistic behavior [7]. Virtue and care ethics helps the learner to utilize “internalized values,” and incorporates emotion with cognition and complements other ethics teaching [15]. Promoting virtue calls for sound role-modeling by educators [14].

The Care as a Virtue theme addresses the concept of caring as being an augmenting factor to virtue ethics by incorporating emotion [11]. With this perspective, care helps to reveal the type of humanistic practices that learners might strive for, and how education should focus on caring to amplify the healing process [11]. The relationship of care to medicine is thought to be conveyed by way of love, connection, and caring through empathy [22]. Being a caring person was cited as an important attribute of highly influential educators of clinicians [6]. Often, care was coupled with other virtues such as altruism, compassion, and empathy in its explication in promoting humanism [20].

The theme of Dissonance/Virtue versus Principle describes how traditional deontological and consequentialist approaches to ethics in medical school can conflict with virtue and care ethics frameworks in such a way that they may seem to ignore or overlook the value of character or caring relationships among those with unequal power within medical ethics [16]. Some call for a need to segregate the teaching of principles in medical ethics from aspects of virtue and care ethics [14]. Concepts of care and character are very different in the minds of students than the notion of duties and obligations [15]. Virtue ethics focuses on how character promotes traits that allow a person to be a better healer whereas respect for autonomy is concerned with what one owes to another person as a right [16]. Some argue that virtue ethics is a better means for analyzing ethical issues in healthcare than Principlism, leading to better acquisition of humanistic behavior [20]. The complementary nature of these teachings to one another is cited to augment each other [22]. It has been suggested that learners ought to be taught how to identify and clarify the differences between rules and character [21].

The themes of Pedagogy and Role Modeling describe how the learner needs to be familiarized with virtue and care ethics in healing with both knowledge and learning through observing. With the Pedagogy theme, some papers focus on the educational modalities such as utilizing virtues (specifically relating to roles) in ethical cases to improve understanding [24]. Some are specific on how to teach virtue ethics with extrapolations from nonmedical school environments to healthcare [17]. As noted previously, some believe that teaching by way of virtue ethics is better than that utilizing Principlism, and the former ought to be part of the medical curriculum [20]. This advocacy includes utilizing digital technologies to teach virtue ethics in healthcare environments [2]. Another suggested utilizing teaching strategies using care ethics to shift the focus from inner qualities to relational capacities, incorporating emotions and reason, promoting humanistic healing [11]. With the Role Modeling Theme, papers emphasized the essential aspect of role modelling to promote virtue and care ethics. To enhance virtue, improved role models are needed as well as literature that promotes examples of role models [14]. Narratives can help improve understanding of caring role modelling [6].

The theme of Relationships describes how virtue/care ethics are based upon the character-based interaction of two or more persons, although care ethics emphasizes the inequality of the relationship-based interaction. This theme notes how relationships foster care in medicine through humanistic practice in the caring endeavor [22]. Each practitioner needs to acknowledge how the enhancement of virtue ethics within oneself promotes the betterment of the patient [11]. Virtue and care may effectively be engaged through relationships not only with patients but also with their families to enhance compassion [7]. Patient-centered care implicitly requires a caring attitude towards patients. The role modeling relationship with teachers is thought to support this ethic [3, 14]. Humanistic teachers are influential in imparting care to their learners, and learners are drawn to them as role models of empathy and interpersonal communication [3]. Mentorship enhances care beyond one’s medical training [8]. The relational nature of caring also emphasizes interpersonal aspects of emotion and competency [11]. Educational opportunities are recommended to promote humanism and professionalism to address challenging relationships with patients [14].

The theme of Praxis/Humanistic Behavior concerns the ways in which the learner may come to manifest humanistic behavior when incorporating the needed virtues and care in healing. Perhaps first described by Peabody nearly a century ago [48], virtue and care ethics act as a foundation to humanistic behavior and professionalism in patient care, promoting practice care particularly when achieved with clearly articulated objectives [11]. Outcomes include clarification of values and diversity of values and personal growth, promotion of caring attitudes, and meaningfulness of professionalism as valued endpoints [3, 19]. The types of identified humanistic behavior in these 25 papers included: virtues of compassion, altruism, and self-awareness, and role-modeling by medical educators. These articles included such endpoints to evaluate humanism and professional identity formation as visual narratives that convey humanistic insights [1], Humanistic Practices Teaching Effectiveness Questionnaire [3], narrative-based professionalism portfolios [13], moral reasoning assignments [18], experience sampling methodology [20], how people act in experimental settings [20], and e-portfolios [8]. Other outcome attributes such as mindfulness, exceptional communication skills, and passion for care were also cited [8]. Additionally, there was a focus on how virtues such as compassion can be nurtured and thereby enhance the educational process and facilitate humanistic care to patients and their families [7].

The theme of Culture/Hidden Curriculum addresses how deleterious aspects of medical culture (through damaging moral conduct and toxic character) can upend humanistic educational efforts [15]. This theme describes the challenges of an adverse culture that can be hostile to virtue and care ethics. Medical training can be seen as a negative reinforcer of empathetic virtue [12]. As a result, there can be a noteworthy gap between the virtuous physician one aspires to be and the physician that one is, requiring that we incorporate aspects of pedagogy and role modeling to improve professional identity formation in recognizing the need for cultural change [14]. There can be many challenges in promoting virtue and care ethics given that we are human, so we must address the moral distress that occurs when we fall short of our aspirations [23].

The identified noteworthy considerations of professionalism, narrative, burnout prevention, and learning communities, can be interpreted as additional means to counter impediments that adversely influence the cultivation of humanistic behavior. Professionalism is relevant in this scoping review as the aspirational intent of virtue and care ethics are professional development to overcome the detractive aspects of medical education that negatively affect behavior. Professionalism is cited by many papers as an application of virtue ethics to the medical social contract, based on altruism and contract keeping [12]. Reflective exercises have been utilized to promote care and caring, resilience and wellness with the goal of becoming humanistic physicians [8]. Virtue ethics is seen as not only a basis for professionalism but also a means to professional formation [14]. Virtue ethics is seen as a means by which to foster ethical and professional educators [16]. The relationship between virtue ethics and professionalism helps to guide the learner highlighting the aspirations of character towards one’s own moral development [23]. As there is an educational requirement for professionalism, cultivating appropriate virtue and care ethics promote patient care [16]. One paper cites “professional responsibility” as a virtue, playing a central role in the development of medical professionalism [10]. Compassionate care and professional identity that is respectful of patients promotes humanism, which is vital to the professionalism enterprise [14].

The consideration of Narrative describes the modality of teaching using narratives on physician virtues, motivations, and behavior looking at literature (rather than physician-life stories) to encourage compassion [13]. Others advocate personal medical narratives to enhance humanistic care [1], using stories told from the perspective of physicians and then applying a virtue framework to better understand the ethical aspects of character [25]. Narrative-based professionalism efforts are intended to promote integrity, empathy, and patient engagement while promoting compassionate and responsive professional identity [14]. Personal narrative as a reflective writing exercise has been used to enhance student resilience and improve mindfulness [8]. Another avenue of narrative has been the use of appreciative inquiry in writing narratives about influential teachers to promote excellence, self-awareness, encouragement, and role modeling [6].

The consideration of Prevention of Burnout specifically focuses on burnout prevention by focusing on relationships with patients, open discussions on burnout, and the challenges of suffering patients [5]. Learning Communities have been utilized to enhance role modelling of humanism and convey caring attitudes as part of faculty development across several medical schools [3]. That utility includes requisite faculty development to enhance mindfulness, humanism, and communication skills, as well as to concentrate on reflective learning in groups and enhance skill building and role modeling [6]. With medical students, efforts to foster altruism and compassion can enhance care and humanism toward patients [7].

When the articles were evaluated for relevant metaphors (Table 3), these cluster into two domains: Aspirational and Detractive. The Aspirational metaphors consist of care and virtue being “Habits of the heart,” [18], that virtue was like “A beacon on a map,” [23] and that one needed to push oneself as if “Learning to drive on the edge of the tires” [11]. The Detractive metaphors consist of how physicians can see themselves versus others as “Heroes vs. Villains,” that physicians believe that they are in an “Ailing culture” and that narrative can assist in exploring the “gap between virtue and what we do” [13]. The utilization of ethics can be seen as a “Bludgeon,” [12] and that the journey of becoming and being a physician can be one of “Medical Monasticism” (i.e., self-sacrifice) [23]. This analysis shows a deep tension in the literature on virtue and care ethics education. These thematic and metaphorical elements of the articles that we reviewed lend support to the idea, discussed below, that within the medical curriculum the efficacy of (and desire for) virtue and/or care ethics education is significantly tempered by systemic issues (especially mastery of medical information and procedures), which could hinder virtue development and role-modeling.

**Table 3:** Relevant Metaphors

Aspirational	Detractive
“Habits of the heart”	“Heroes vs. Villains” (Physicians vs. others)
“A beacon on a map” (Virtue)	“Ailing culture”
“Learning to drive on the edge of the tires”	“Bludgeon” of ethics
	“Medical Monasticism” (Self-Sacrifice)

## Discussion

This scoping review is innovative with a specific goal of identifying thematic connections between virtue and care ethics, medical education, and humanistic clinical practice. While virtue ethics goes back to the time of Plato and Aristotle [49], its Renaissance and then development within contemporary bioethics and inclusion into medical education has been going on for half a century. Further, care ethics, has been conceptualized over the last 40 years, and highlights “the relational, the local and the particular” [53]. As an ethical framework, its central concern is not the development of a systematic moral theory with generalizable rules and duties but at its core emphasizes human interdependence and inequality in caring relationships [31]. Despite the robust literature on these approaches to ethics, there has been a relative lack of articles that build upon aspects of virtue and/or care ethics towards health care curricular inclusion with an endpoint of humanistic behavior. What was learned in this study is that medical educators do have a small body of work that they can access to include these aspects of humanism in medical education [37]. This review had a noted limitation of a constricted size of citations, such that full text searching of abstracts was necessary (which reflects the late emergence of virtue and care ethics in medical education only over the past three decades). Also, we relied upon authors using the term *humanism, care ethics*, and *virtue ethics* so that manuscripts could be discovered in our search. Of note, when we attempted to use *professionalism*, it did not alter our results. Our resultant list of citations was from reputable journals of medical education and ethics and worth consideration for future curricular development. What we can hope for now is a growth of more peer-reviewed articles that flesh out understanding of virtue and care ethics and recognize their differences. That is, virtue ethics has a focus on the individual character of the physician, whereas care ethics highlights interdependence, which may foster communities of practice to support resilience and personal growth [32]. Such medical education curricula may utilize endpoints that bolster the professional growth of future physicians such that they will be the exemplars of humanistic care. Identifying useful methods of measuring or assessing humanistic care, beyond self-report, will be important to evaluate the resultant curricula. Further, this project may prove facilitative in the construct of model curricula in virtue and care ethics education to enhance learner humanism by using these and similar articles as a pedagogical resource.

## Conclusion

This scoping review set out to evaluate publications in the medical literature that address aspects of virtue and care ethics in medical curricula toward the cultivation of learner humanism. We successfully found twenty-five papers that were relevant, of which eight had full implementation through curricula. The themes noted assist in understanding the many factors that play a role in using care and virtue ethics in curricula toward humanistic behavior. It is the intention of this working group to encourage others to initiate model curricula that play off the strengths of the results of this paper to translate virtue and care ethics into a curriculum that promotes humanistic behavior. These published resources and the present analysis of them may serve as a foundation for sound curriculum related to humanism. Implementation will assist in utilizing virtue and care ethics and promoting humanism among physicians.

## Data Availability

The datasets used and/or analyzed during the current study are available from the corresponding author on reasonable request.
